# A comparison of central nervous system involvement in patients with classical Fabry disease or the later-onset subtype with the IVS4+919G>A mutation

**DOI:** 10.1186/s12883-017-0810-9

**Published:** 2017-02-06

**Authors:** Han-Jui Lee, Ting-Rong Hsu, Sheng-Che Hung, Wen-Chung Yu, Tzu-Hung Chu, Chia-Feng Yang, Svetlana Bizjajeva, Chui-Mei Tiu, Dau-Ming Niu

**Affiliations:** 1 Department of Radiology, Taipei Veterans General Hospital, Taipei, Taiwan; 2 School of Medicine, National Yang-Ming University, Taipei, Taiwan; 3 Institute of Clinical Medicine, National Yang-Ming University, Taipei, Taiwan; 4 Department of Pediatrics, Taipei Veterans General Hospital, Taipei, Taiwan; 5 Department of Biomedical Imaging and Radiological Sciences, National Yang-Ming University, Taipei, Taiwan; 6 Division of Cardiology, Department of Medicine, Taipei Veterans General Hospital, Taipei, Taiwan; 7 Shire, Zug, Switzerland

**Keywords:** Agalsidase alfa, Central nervous system manifestations, Fabry Outcome Survey, Later-onset Fabry disease, Magnetic resonance imaging

## Abstract

**Background:**

Patients with the later-onset IVS4+919G>A (IVS4) Fabry mutation are known to have positive central nervous system involvement compared with age- and sex-matched controls. This study compares central nervous system manifestations in patients with the IVS4 mutation or classical Fabry mutations.

**Methods:**

This was a retrospective analysis of magnetic resonance imaging (MRI) data from Taiwanese patients enrolled in the Fabry Outcome Survey (sponsored by Shire; data extracted March 2015).

**Results:**

Twenty-five IVS4 (19 males) and 12 (four males) classical Fabry patients underwent MRI at a median (range) age of 60.7 (45.0–70.4) and 43.0 (18.0–61.4) years, respectively. All patients received agalsidase alfa enzyme replacement therapy; two (16.7%) classical Fabry patients underwent MRI before treatment start. The pulvinar sign occurred in eight (32.0%; seven males) IVS4 and six (50.0%; three males) classical Fabry patients. Infarction occurred in eight (32.0%) IVS4 and four (33.3%) classical Fabry patients. Fazekas scores of 0, 1, 2, and 3 were found for 15 (60.0%), seven (28.0%), two (8.0%), and one (4.0%) of the IVS4 patients and for six (50.0%), four (33.3%), two (16.7%), and 0 classical Fabry patients, respectively. Abnormal height bifurcation of the basilar artery was observed in 40.0% of IVS4 and 58.3% of classical Fabry patients; abnormal laterality was observed in 4.0% of IVS4 and 16.7% of classical Fabry patients. Median (range) basilar artery diameter was 2.7 (1.4–4.0) mm in IVS4 and 3.2 (2.3–4.7) mm in classical Fabry patients (*P* = 0.0293); vascular stenosis was noted in 8.3% of IVS4 patients but in no classical Fabry patients.

**Conclusions:**

A similar range of MRI findings was found for both IVS4 and classical Fabry patients. Notably, basilar artery diameter was larger in classical Fabry patients than IVS4 patients.

## Background

Fabry disease (FD, MIM 301500) is an X-linked lysosomal disorder resulting from lysosomal α-galactosidase A deficiency, which subsequently leads to the accumulation of glycosphingolipids, primarily globotriaosylceramide, throughout the body [1]. This multisystemic disorder commonly manifests in childhood or adolescence with symptoms including acroparesthesia, cornea verticillata, and gastrointestinal complaints [2]. Progression to renal failure, hypertrophic cardiomyopathy, and cerebrovascular complications occurs in later life; these comprise the leading causes of premature death in FD [3]. Organ failure attributed to FD can reduce life expectancy by approximately 25 years in males [4] and 15 years in females [5]. Enzyme replacement therapy (ERT) can slow disease progression [6] and, if started early enough, may even be able to prevent irreversible organ damage.

Neurological signs and symptoms are commonly reported in FD and can start during childhood or adolescence [7]. Neuropathic pain, vertigo, tinnitus, stroke, and transient ischemic attack are some of the commonly reported neurological features of FD [8, 9]. White matter lesions and infarction, increased signal intensity in the lateral pulvinar (known as the pulvinar sign), and increased diameter and tortuosity of the basilar artery [8, 10–12] are some of the abnormalities that have been observed upon brain magnetic resonance imaging (MRI) in patients with FD. Increased diameter of the basilar artery and presence of the pulvinar sign are reportedly useful in FD diagnosis, particularly when found alongside other, less specific, neurological findings that are also known to occur in FD [10–12].

The incidence of FD ranges from 1 in 40,000 to 1 in 117,000 live births in the general population [1, 13]. There are 2 major phenotypes of FD, “classical” (type 1) and “later-onset” (type 2) subtypes [14]. In Taiwan, the Chinese hotspot IVS4+919G>A (IVS4) mutation occurs at a high frequency [15, 16] and is reported to be a pathogenic, later-onset, cardiac-specific Fabry mutation [17]. Despite its high frequency, the pathology and neurological complications of FD in individuals with the later-onset IVS4 mutation currently are not well understood. In our previous comparison of patients carrying the IVS4 mutation with healthy age- and sex-matched controls, we found a greater frequency of infarctions (35% vs 0%; *P* = 0.001) and the pulvinar sign (30% vs 0%; *P* = 0.002), and a greater volume of white matter hyperintensities (1.1583 cm^3^ vs 0.1354 cm^3^; *P* = 0.004) in patients with IVS4-type FD [18]. The objective of the current study is to retrospectively review and compare the severity of central nervous system manifestations in Taiwanese patients with the later-onset IVS4 mutation or classical Fabry mutations.

## Methods

### Study design

This was a retrospective analysis of MRI data from Taiwanese patients enrolled in the Fabry Outcome Survey (FOS; sponsored by Shire Human Genetic Therapies, Inc). At the Taipei Veterans General Hospital (TVGH), brain imaging is routinely performed for patients with FD. The protocol for white matter (fluid-attenuated inversion recovery [FLAIR]) and cerebral artery (MR angiography) imaging has been optimized but not restricted to the same machine; thus, patients receive similar imaging protocols but can be randomly assigned to different scanners. This study analysed MRI and neurological signs and symptoms data gathered at the TVGH along with demographics and baseline data collected in FOS for the same patients. The FOS registry collates outcomes data from patients with confirmed FD who are receiving, or are eligible for, ERT with agalsidase alfa. Treated patients receive agalsidase alfa 0.2 mg/kg body weight every other week. Inclusion criteria for this analysis were being Taiwanese, presence of the Chinese hotspot IVS4 mutation or classical Fabry mutations, as confirmed by molecular analysis, and having undergone brain MRI at any point after FD diagnosis. The data for this analysis were extracted from the FOS database in March 2015.

The Institutional Review Board of TVGH approved participation in FOS and the MRI analysis, and all patients gave written informed consent before their data were entered into the FOS database. Two board certified neuroradiologists, who were blinded to the type of disease, reviewed the brain MRIs from all patients by consensus.

### Brain imaging

Non-contrast MRIs were obtained on one of three 1.5-tesla or 3-tesla scanners (Signa Excite 1.5T, GE Healthcare, Milwaukee, WI, USA; SignaHDxt 1.5T, GE Healthcare; Discovery MR750 3.0T, GE Healthcare). Axial spin-echo T1-weighted imaging parameters were 1.5T, 600–700/8–11/2 (TR/TE/NEX) and 3T, 360/9/1 (TR/TE/NEX). Axial FLAIR imaging parameters were 1.5T, 9000/92/2250 ms (TR/TE/TI) and 3T, 9000/145/2250 ms (TR/TE/TI). Time-of-flight angiography parameters were 1.5T, 26–28/6.8 ms (TR/TE) and 3T, 25/2.8 ms (TR/TE); flip angle 20°, voxel size 0.4 x 0.4 x 0.5 mm.

### Qualitative assessment

The methods used for image analysis are described in our previous study [18]. The presence and location of infarction was categorized as none, anterior circulation alone, posterior circulation alone, or both anterior and posterior circulation. Furthermore, high signal changes on T1-weighted images bilaterally at the lateral pulvinar were considered as the pulvinar sign [11].

### Semi-quantitative assessment

Deep white matter hyperintensities on T2-weighted images or FLAIR sequences were graded according to the Fazekas scale, which classifies white matter hyperintensities according to the following scoring system: 0, absent; 1, punctate foci; 2, beginning of confluence of foci; and 3, large confluent areas [19].

The degree of elongation and tortuosity of the basilar artery were evaluated using Smoker’s criteria [20] according to the height of basilar artery bifurcation and its most lateral position. The scale used to categorize the height of basilar artery bifurcation was as follows: 0, at or below the dorsum sellae; 1, within the suprasellar cistern; 2, at the level of the floor of the third ventricle; and 3, indenting and elevating the floor of the third ventricle. The most lateral position of the basilar artery was graded as follows: 0, midline throughout; 1, medial to lateral margins of the clivus or dorsum sellae; 2, lateral to lateral margins of the clivus or dorsum sellae; and 3, situated in the cerebellopontine angle cistern.

### Quantitative assessment

The diameter of the basilar artery was measured on a workstation (AZE Virtual Place Plus, AZE Ltd., Tokyo, Japan) by an observer who was blinded to all clinical information. A line was drawn perpendicular to the middle portion of the basilar artery on the sagittal view of a three-dimensional time-of-flight magnetic resonance angiogram at the maximum intensity projection. The observer then recorded the diameter as the full width at half maximum of this middle segment. Vascular stenosis was identified when the diameter of the basilar artery was less than 2 mm or appeared hypoplastic.

### Neurological signs and symptoms

The prevalence of neurological signs and symptoms was obtained from the FOS database and compared between patients with IVS4 or classical Fabry mutations and MRI data. The neurological signs and symptoms analysed include stroke, sudden onset of numbness or weakness in the extremities, asymmetric facial expression, dysarthria, sudden onset of blurred vision or diplopia, depression, emotional or personality changes, forgetfulness, tinnitus, and vertigo.

### Statistical analysis

The statistical analysis was performed using SAS software, version 9.2 (SAS Institute Inc., Cary, NC, USA). Descriptive statistics were calculated for demographic, MRI, and signs and symptoms data. For continuous variables, 95% confidence intervals for means were calculated using t-distribution, and for binary variables exact (Clopper-Pearson) 95% confidence intervals were computed. Differences between IVS4 versus classical Fabry mutations in binary outcome variables were assessed using Fisher’s exact test. Differences in continuous variables between patients with IVS4 versus classical Fabry mutations were evaluated using the Wilcoxon rank-sum test, either exact (when computationally feasible) or normal approximation. Potential associations between mutation type and presence of neurological signs and symptoms were examined using Fisher’s exact test; estimated odds ratios together with 95% exact confidence limits are reported. All statistical analyses are exploratory and the results of the statistical tests (*p*-values) are interpreted descriptively, as hypothesis generation rather than hypothesis testing. The level of significance was set to 5% without any multiplicity adjustment. *P*-values above the significance level of 5% are not considered as confirmation of no difference between the groups, and *p*-values below 5% are not considered as confirmation of a difference between the groups.

## Results

A total of 37 Taiwanese patients registered in FOS had brain MRI data; of these, twice as many had the IVS4 mutation (67.6%) than classical Fabry mutations (32.4%). The majority of IVS4 patients were male (76.0%) whereas females comprised the majority of classical Fabry patients (67.0%; Table 1). Median age at symptom onset, diagnosis, FOS entry, and MRI assessment was greater for IVS4 patients than classical Fabry patients (Table 1). All patients received ERT with agalsidase alfa; all IVS4 patients and 83.3% of classical Fabry patients underwent brain MRI after ERT initiation (Table 1).

**Table 1:** Demographic characteristics of Taiwanese patients with MRI data registered in FOS as of March 2014

Characteristic	IVS4 mutation *n* = 25	Classical Fabry mutations *n* = 12	*P*-value
Sex, *n* (%)			
Male	19 (76.0)	4 (33.0)	0.0274^a^
95% CI	0.55-0.91	0.10-0.65	
Female	6 (24.0)	8 (67.0)	
Age at symptom onset, years^c^			
Mean (SD)	50.3 (7.8)	9.5 (2.0)	
95% CI	46.2-54.5	8.1-10.9	
Median (range)	48.0 (38.0-65.0)	10.0 (6.0-12.0)	<0.0001^b^
Age at diagnosis, years			
Mean (SD)	57.6 (6.9)	39.8 (14.5)	
95% CI	54.7-60.4	30.6-49.1	
Median (range)	59.0 (42.0-67.0)	40.0 14.0-60.0)	0.0001^b^
Age at FOS entry, years			
Mean (SD)	59.7 (6.7)	42.4 (14.1)	
95% CI	56.9-62.5	33.4-51.4	
Median (range)	60.3 (44.8-69.7)	43.3 (17.4-61.2)	<0.0001^b^
Received ERT, *n*			
Yes	25	12	NA
95% CI	0.86-1.00	0.74-1.00	
Age at treatment start, years			
Mean (SD)	58.6 (6.8)	40.9 (14.5)	
95% CI	55.8-61.5	31.7-50.1	
Median (range)	59.7 (44.1-68.3)	40.9 (14.9-61.3)	0.0001^b^
MRI after treatment initiation, *n* (%)			
Yes	25 (100.0)	10 (83.3)	0.0991^a^
95% CI	0.86-1.00	0.52-0.98	
Age at MRI assessment, years			
Mean (SD)	60.0 (6.8)	42.6 (14.3)	
95% CI	57.2-62.8	33.6-51.7	
Median (range)	60.7 (45.0-70.4)	43.0 (18.0-61.4)	0.0001^b^

*ERT*, enzyme replacement therapy; *FOS*, Fabry Outcome Survey; *IVS4*, IVS4+919G>A; *MRI*, magnetic resonance imaging; *NA*, not available

^a^Fisher’s exact test

^b^Wilcoxon rank-sum test

^c^Data missing from 9 IVS4 (*n* = 16) and 2 classical FD (*n* = 10) patients

**Fig. 1:**
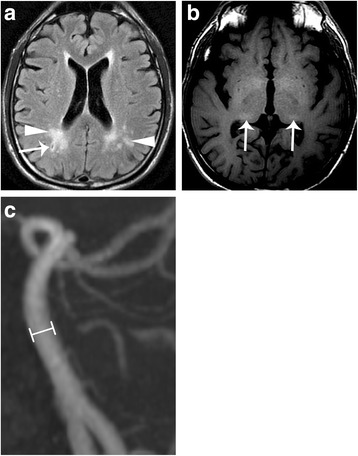
A middle-aged man with classical-type Fabry disease who suffered from chronic renal disease. **a** Brain magnetic resonance axial T2 fluid-attention inversion recovery reveals an old lacunar infarct at the right centrum semiovale (arrow) and deep white matter hyperintensities (arrowheads). **b** Axial T1-weighted image reveals high signal changes at the bilateral posterior thalamus (arrows), the pulvinar sign. **c** The diameter at the middle segment of the basilar artery (lines) was measured as 4.0 mm on a three-dimensional time-of-flight magnetic resonance angiogram

**Fig. 2:**
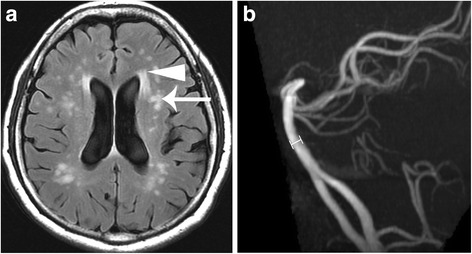
A middle-aged female patient with IVS4+919G>A-type Fabry disease who suffered from hypertrophic cardiomyopathy and sudden onset of limb weakness. **a** Brain magnetic resonance axial T2 fluid-attention inversion recovery shows old lacunar infarct at the left corona radiata (arrow) and increased deep white matter hyperintensities (arrowhead). **b** The diameter at the middle segment of the basilar artery was measured as 3.3 mm on a three-dimensional time-of-flight magnetic resonance angiogram

**Fig. 3:**
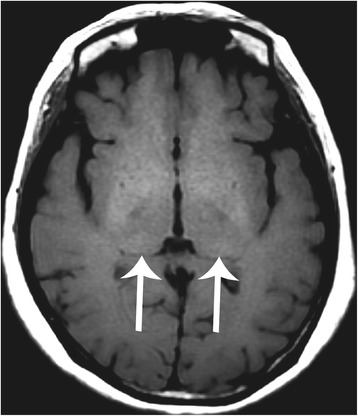
A middle-aged female patient with IVS4+919G>A-type Fabry disease with hypertrophic cardiomyopathy. Brain magnetic resonance axial T1-weighted image reveals the pulvinar sign with high signal changes at the bilateral posterior thalamus (arrows)

Brain MRI in both IVS4 and classical Fabry patients revealed infarction, deep matter hyperintensities (Fig. 1, Fig. 2), and the pulvinar sign (Fig. 1, Fig. 3). Overall, infarcts were observed in similar proportions of IVS4 and classical Fabry patients (32.0% and 33.3%, respectively; Table 2), although with some differences in the site of occurrence. Anterior circulation stroke alone and posterior circulation stroke alone each occurred in 8.0% of IVS4 patients, and both anterior and posterior circulation stroke occurred in 16.0% of IVS4 patients. Posterior circulation stroke alone was not observed in classical Fabry patients; instead, anterior circulation stroke alone and both anterior and posterior circulation stroke each occurred in 16.7% of classical Fabry patients (Table 2). Hemorrhage was noted in MRIs from 16.7% of classical Fabry patients, but not in any MRIs from IVS4 patients (Table 2).

**Table 2:** MRI and MRA findings in patients with IVS4 versus classical Fabry mutations

Parameters	IVS4 mutation *n* = 25	Classical Fabry mutations *n* = 12	*P*-value
MRI findings			
Pulvinar sign, *n* (%)	8 (32.0)	6 (50.0)	0.4701^a^
95% CI	0.15-0.54	0.21-0.79	
Infarct, *n* (%)	8 (32.0)	4 (33.3)	0.6759^b^
95% CI	0.15-0.54	0.10-0.65	
Anterior circulation stroke	2 (8.0)	2 (16.7)	
Posterior circulation stroke	2 (8.0)	0	
Both anterior and posterior circulation stroke	4 (16.0)	2 (16.7)	
None of them	17 (68.0)	8 (66.7)	
Hemorrhage (microbleeds), *n* (%)	0	2 (16.7)	0.0991^a^
Fazekas score, *n*	25	12	0.7378^a^
0: No or a single punctate WM lesion	15 (60.0)	6 (50.0)	
1: Multiple punctate WM lesions	7 (28.0)	4 (33.3)	
2: Beginning of lesion confluence	2 (8.0)	2 (16.7)	
3: Large confluent lesion	1 (4.0)	0	
Height bifurcation of the BA, *n*	25	12	0.4276^*^
0: At or below the dorsum sellae	6 (24.0)	2 (16.7)	
1: Within the suprasellar cistern	9 (36.0)	3 (25.0)	
2: At the third ventricle floor	10 (40.0)	6 (50.0)	
3: Indenting and elevating the third ventricle floor	0	1 (8.3)	
Abnormal height bifurcation	10 (40.0)	7 (58.3)	
Laterality of the BA, *n*	25	12	0.3652^a^
0: Midline throughout	12 (48.0)	4 (33.3)	
1: Medial to lateral margins of clivus or dorsum sellae	12 (48.0)	6 (50.0)	
2: Lateral to lateral margins of clivus or dorsum sellae	1 (4.0)	2 (16.7)	
3: In the cerebellopontine angle cistern	0	0	
Abnormal laterality	1 (4.0)	2 (16.7)	0.2407^a^
95% CI	0.00-0.20	0.02-0.48	
MRA findings			
BA diameter, mm			
Mean (SD)	2.7 (0.6)	3.3 (0.7)	
95% CI	2.5-3.0	2.9-3.8	
Median (range)	2.7 (1.4-4.0)	3.2 (2.3-4.7)	0.0293^b^
Vascular stenosis/BA hypoplasia, *n* ^c^ (%)	2 (8.3)	0	0.5429^a^
95% CI	0.01-0.27	0.74-1.00	

*BA*, basilar artery; *IVS4*, IVS4+919G>A; *MRA*, magnetic resonance angiography; *MRI*, magnetic resonance imaging; *WM*, white matter

^a^Fisher’s exact test

^b^Wilcoxon rank-sum test

^c^Data missing from one IVS4 patient, thus *n* = 24

Fazekas scores for deep white matter hyperintensities were assigned for 100.0% of both IVS4 and classical Fabry patients. In each group, the largest proportion of patients was assigned a Fazekas score of 0 (60.0% for IVS4 and 50.0% for classical Fabry patients), followed by scores of 1 (28.0% and 33.3%), 2 (8.0% and 16.7%), and 3 (4.0% IVS4 patients only; Table 2). The pulvinar sign was observed in a greater proportion of classical Fabry patients (50.0%) than IVS4 patients (32.0%; Table 2).

Abnormal height bifurcation of the basilar artery was observed in 40.0% of IVS4 and 58.3% of classical Fabry patients (at the third ventricular floor in all IVS4 and 50.0% of classical Fabry patients, and indenting and elevating the third ventricular floor in 8.3% of classical Fabry patients; Table 2). Abnormal laterality of the basilar artery was observed in 4.0% of IVS4 and 16.7% of classical Fabry patients (lateral to lateral margins of the clivus or dorsum sellae in all patients with abnormal laterality; Table 2).

The median diameter of the middle segment of the basilar artery was larger in classical Fabry patients than it was in IVS4 patients (3.2 mm vs 2.7 mm; *P* = 0.0293; Table 2). Vascular stenosis of the basilar artery was noted in 8.3% of IVS4 patients but in none of the classical Fabry patients (Table 2).

Of the IVS4 patients who did not demonstrate MRI evidence of infarction, eight of 17 (47.1%) presented with one or more non-specific neurological symptoms, including numbness/weakness (*n* = 1), depression (*n* = 1), forgetfulness (*n* = 2), tinnitus (*n* = 2), vertigo (*n* = 4; Table 3). For IVS4 patients with MRI evidence of infarction, six of eight (75.0%) presented with one or more non-specific neurological symptoms, including numbness/weakness (*n* = 2), asymmetric face (*n* = 1), blurred vision/diplopia (*n* = 1), emotional change (*n* = 2), personality change (*n* = 1), forgetfulness (*n* = 4), tinnitus (*n* = 1), and vertigo (*n* = 1), and two patients were clinically silent. Of the classical Fabry patients who showed no MRI evidence of infarction, four of eight (50.0%) reported neurological complaints, including numbness/weakness (*n* = 1), emotional change (*n* = 3), forgetfulness (*n* = 1), and tinnitus (*n* = 1). Of the four classical Fabry patients with MRI evidence of infarction, two reported symptoms of numbness/weakness, one of whom also reported stroke/minor stroke and dysarthria, and the other two were clinically silent. No statistically significant associations were found between the type of mutation (IVS4 or classical Fabry) and any neurological sign or symptom.

**Table 3:** Neurological signs and symptoms in patients with IVS4 or classical Fabry mutations

Neurological sign or symptom, *n* (%)	IVS4 mutation *n* = 25	Classical Fabry mutations *n* = 12	Odds ratio (exact 95% CI); *P*-value	No MRI evidence of infarction		MRI evidence of infarction	
				IVS4 mutation *n* = 17	Classical Fabry mutations *n* = 8	IVS4 mutation *n* = 8	Classical Fabry mutations *n* = 4
Any neurological manifestation	14 (56.0)	6 (50.0)	1.27 (0.26-6.28); 1.0000	8 (47.1)	4 (50.0)	6 (75.0)	2 (50.0)
Stroke/minor stroke	1 (4.0)	1 (8.3)	0.46 (0.01-39.22); 1.0000	0	0	1 (12.5)	1 (25.0)
Numbness/weakness	3 (12.0)	3 (25.0)	0.41 (0.05-3.74); 0.3666	1 (5.9)	1 (12.5)	2 (25.0)	2 (50.0)
Asymmetric face	1 (4.0)	0	(0.03-NA); 1.0000	0	0	1 (12.5)	0
Dysarthria	0	1 (8.3)	(0.03-NA); 0.3243	0	0	0	1 (25.0)
Blurred vision/diplopia	1 (4.0)	0	(0.03-NA); 1.0000	0	0	1 (12.5)	0
Depression	1 (4.0)	0	(0.03-NA); 1.0000	1 (5.9)	0	0	0
Emotional change	2 (8.0)	3 (25.0)	0.26 (0.02-2.79); 0.3035	0	3 (37.5)	2 (25.0)	0
Personality change	1 (4.0)	0	(0.03-NA); 1.0000	0	0	1 (12.5)	0
Forgetfulness	6 (24.0)	1 (8.3)	3.47 (0.34-174.06); 0.3891	2 (11.8)	1 (12.5)	4 (50.0)	0
Tinnitus	3 (12.0)	1 (8.3)	1.50 (0.10-85.80); 1.0000	2 (11.8)	1 (12.5)	1 (12.5)	0
Vertigo	5 (20.0)	0	(0.62-NA); 0.1521	4 (23.5)	0	1 (12.5)	0

*IVS4*, IVS4+919G>A; *MRI*, magnetic resonance imaging; *NA*, not available

## Discussion

Fabry disease is classified as the classical phenotype (type 1) or the later-onset phenotype (type 2), which tends to have mutation-specific renal or cardiac damage. Classical FD is characterized by frequent central nervous system involvement, which may be caused by the deposition of glycosphingolipids in cerebrovascular endothelial cells, a consequence of cardiogenic embolism from cardiomyopathy, valvular heart disease, ischemic heart disease, and/or arrhythmias [21–24]. Cardiac involvement is already acknowledged in later-onset IVS4 FD, but the degree of extra-cardiac involvement is still not well known. Brain MRI findings in our previous study demonstrated a greater degree of neurological involvement in patients with IVS4 FD compared with age- and sex-matched healthy controls [18]. In this study, we showed that patients carrying the later-onset IVS4 mutation had similar central nervous system involvement to that of classical Fabry patients, but with a lower degree of basilar artery dilatation.

In this comparison between patients with IVS4 and classical FD, the prevalence of infarction was similar in each group (32.0% for IVS4 patients and 33.3% for classical Fabry patients). The incidence of stroke found in our patients is much higher than in the general population [25], but similar to the results of other studies on FD [21, 22].

Vertebrobasilar dolichoectasia is thought to be an early and frequent sign of classical FD [26], and tends to be more frequently observed in elderly or male FD patients [18, 26]. In our study, median basilar artery diameter was larger in classical Fabry patients than IVS4 patients, despite the IVS4 group containing more males and having an older median age than the classical Fabry group. This may reflect less basilar artery involvement in IVS4 patients than classical Fabry patients. The mechanism of dolichoectasia is still not completely understood. In contrast with the histology of classical FD vasculopathy, and despite significant globotriaosylceramide accumulation in cardiomyocytes, no endothelial deposition of globotriaosylceramide was noted in our previous myocardial biopsy study [17], or in other reports on later-onset, cardiac-specific variants [27–29]. These findings support the theory that later-onset FD should be considered a unique entity that is different from classical FD, owing to the primary involvement of cardiomyocytes instead of endothelial cells. Furthermore, later-onset FD presentation and course might not be the same as typically observed in classical FD. Thus, long-term follow-up studies of IVS4 FD are needed for a more thorough understanding of the progression of this disease.

White matter hyperintensities, which are thought to be related to small vessel disease and secondary to the severity of perfusion dysfunction in FD cerebral vasculopathy, were quite similar in frequency between the two groups [30]. Furthermore, the pulvinar sign, which may be caused by regional hyperperfusion and reflect associated subtle dystrophic calcifications and end-organ damage [10, 11], was found in 32% and 50% of patients in the IVS4 and classical FD groups. This might provide supportive evidence of CNS involvement in IVS4 FD. However, the median age of the IVS4 group is higher than the classical Fabry group and patient numbers are too small to provide a solid conclusion; thus, further investigation with a larger population is necessary.

In addition to cerebrovascular events, cognitive dysfunction, although mild in nature, and depression are already known in FD patients [31]. In the current analysis, in terms of neurological presentations, IVS4 and classical Fabry patients shared several similarities. First, as previously mentioned, the observed incidence of infarction was similar in the two groups. Silent infarcts are commonly noted events in FD, but the frequency of silent brain infarcts in FD is still not yet known [32]. Second, there was a high prevalence of non-specific signs and symptoms or silent infarcts in both groups (62.5% in IVS4 patients and 50.0% in classical Fabry patients). These findings indicate that regular neurological and neuroimaging assessments are important and should be recommended for patients with the cardiac IVS4 mutation.

This study had some limitations that should be addressed. First, the FOS registry is not designed to confirm differences between groups of patients and because of the small group sizes the statistical tests lacked power and robustness, restricting the analyses to the descriptive level. Second, differences were observed in age at MRI assessment and in the proportion of females in each group; thus, as the incidence of stroke among patients with FD increases with age [21], the results herein must be interpreted with caution. A large longitudinal cohort comparison is needed to effectively evaluate neurological differences between patients with IVS4 and classical Fabry mutations.

## Conclusions

In conclusion, the range of abnormalities found on brain MRI for classical Fabry patients is consistent with previous observations and also is similar in patients with the IVS4 mutation. Patients with the IVS4 mutation experience similar neurological signs and symptoms to patients with classical Fabry mutations.

## Abbreviations

BA: Basilar artery
ERT: Enzyme replacement therapy
FD: Fabry disease
FLAIR: Fluid-attention inversion recovery
FOS: Fabry Outcome Survey
IVS4: IVS4+919G>A
MRA: Magnetic resonance angiography
MRI: Magnetic resonance imaging
WM: White matter

## References

[1] Desnick RJ, Ioannou YA, Eng CM, Scriver CR, Beaudet AL, Sly WS, Valle D (2001). α-Galactosidase a deficiency: Fabry disease. The metabolic and molecular basis of inherited disease.

[2] Ramaswami U, Wendt S, Pintos-Morell G, Parini R, Whybra C, Leon Leal JA, Santus F, Beck M (2007). Enzyme replacement therapy with agalsidase alfa in children with Fabry disease. Acta Paediatr.

[3] Mehta A, Widmer U, Mehta A, Beck M, Sunder-Plassmann G (2006). Natural history of Fabry disease. Fabry disease: perspectives from 5 years of FOS.

[4] Schiffmann R, Warnock DG, Banikazemi M, Bultas J, Linthorst GE, Packman S, Sorensen SA, Wilcox WR, Desnick RJ (2009). Fabry disease: progression of nephropathy, and prevalence of cardiac and cerebrovascular events before enzyme replacement therapy. Nephrol Dial Transplant.

[5] MacDermot KD, Holmes A, Miners AH (2001). Anderson-Fabry disease: clinical manifestations and impact of disease in a cohort of 60 obligate carrier females. J Med Genet.

[6] Mehta A, Beck M, Elliott P, Giugliani R, Linhart A, Sunder-Plassmann G, Schiffmann R, Barbey F, Ries M, Clarke JT (2009). Fabry Outcome Survey investigators. Enzyme replacement therapy with agalsidase alfa in patients with Fabry’s disease: an analysis of registry data. Lancet.

[7] Mehta A, Ricci R, Widmer U, Dehout F, Garcia de Lorenzo A, Kampmann C, Linhart A, Sunder-Plassmann G, Ries M, Beck M (2004). FOS investigators. Fabry disease defined: baseline clinical manifestations of 366 patients in the Fabry Outcome Survey. Eur J Clin Invest.

[8] Mehta A, Ginsberg L (2005). Natural history of the cerebrovascular complications of Fabry disease. Acta Paediatr Suppl.

[9] Ramaswami U, Parini R, Pintos-Morell G, Kalkum G, Kampmann C (2012). Beck M; FOS Investigators. Fabry disease in children and response to enzyme replacement therapy: results from the Fabry Outcome Survey. Clin Genet.

[10] Moore DF, Ye F, Schiffmann R, Butman JA (2003). Increased signal intensity in the pulvinar on T1-weighted images: a pathognomonic MR imaging sign of Fabry disease. AJNR Am J Neuroradiol.

[11] Takanashi J, Barkovich AJ, Dillon WP, Sherr EH, Hart KA, Packman S (2003). T1 hyperintensity in the pulvinar: key imaging feature for diagnosis of Fabry disease. AJNR Am J Neuroradiol.

[12] Fellgiebel A, Keller I, Martus P, Ropele S, Yakushev I, Bottcher T, Fazekas F, Rolfs A (2011). Basilar artery diameter is a potential screening tool for Fabry disease in young stroke patients. Cerebrovasc Dis.

[13] Meikle PJ, Hopwood JJ, Clague AE, Carey WF (1999). Prevalence of lysosomal storage disorders. JAMA.

[14] Bangari DS, Ashe KM, Desnick RJ, Maloney C, Lydon J, Piepenhagen P, Budman E, Leonard JP, Cheng SH, Marshall J, Thurberg BL (2015). Alpha-galactosidase A knockout mice: progressive organ pathology resembles the type 2 later-onset phenotype of Fabry disease. Am J Pathol.

[15] Lin HY, Chong KW, Hsu JH, Yu HC, Shih CC, Huang CH, Lin SJ, Chen CH, Chiang CC, Ho HJ, Lee PC, Kao CH, Cheng KH, Hsueh C, Niu DM (2009). High incidence of the cardiac variant of Fabry disease revealed by newborn screening in the Taiwan Chinese population. Circ Cardiovasc Genet.

[16] Hwu WL, Chien YH, Lee NC, Chiang SC, Dobrovolny R, Huang AC, Yeh HY, Chao MC, Lin SJ, Kitagawa T, Desnick RJ, Hsu LW (2009). Newborn screening for Fabry disease in Taiwan reveals a high incidence of the later-onset GLA mutation c.936+919G>A (IVS4+919G>A). Hum Mutat.

[17] Hsu TR, Sung SH, Chang FP, Yang CF, Liu HC, Lin HY, Huang CK, Gao HJ, Huang YH, Liao HC, Lee PC, Yang AH, Chiang CC, Lin CY, Yu WC, Niu DM (2014). Endomyocardial biopsies in patients with left ventricular hypertrophy and a common Chinese later-onset Fabry mutation (IVS4+919G>A). Orphanet J Rare Dis.

[18] Lee HJ, Hung SC, Hsu TR, Ko SC, Chui-Mei T, Huang CC, Niu DM, Lin CP. Brain MR imaging findings of cardiac-type Fabry disease with an IVS4+919G>A mutation. AJNR Am J Neuroradiol. 2016. doi:10.3174/ajnr.A4677. 10.3174/ajnr.A4677 PMC7963529 26869469

[19] Fazekas F, Chawluk JB, Alavi A, Hurtig HI, Zimmerman RA (1987). MR signal abnormalities at 1.5 T in Alzheimer’s dementia and normal aging. AJR Am J Roentgenol.

[20] Smoker WR, Corbett JJ, Gentry LR, Keyes WD, Price MJ, McKusker S (1986). High-resolution computed tomography of the basilar artery: 2. Vertebrobasilar dolichoectasia: clinical-pathologic correlation and review. AJNR Am J Neuroradiol.

[21] Sims K, Politei J, Banikazemi M, Lee P (2009). Stroke in Fabry disease frequently occurs before diagnosis and in the absence of other clinical events: natural history data from the Fabry Registry. Stroke.

[22] Buechner S, Moretti M, Burlina AP, Cei G, Manara R, Ricci R, Mignani R, Parini R, Di Vito R, Giordano GP, Simonelli P, Siciliano G, Borsini W (2008). Central nervous system involvement in Anderson-Fabry disease: a clinical and MRI retrospective study. J Neurol Neurosurg Psychiatry.

[23] Rolfs A, Böttcher T, Zschiesche M, Morris P, Winchester B, Bauer P, Walter U, Mix E, Löhr M, Harzer K, Strauss U, Pahnke J, Grossmann A, Benecke R (2005). Prevalence of Fabry disease in patients with cryptogenic stroke: a prospective study. Lancet.

[24] Fellgiebel A, Müller MJ, Ginsberg L (2006). CNS manifestations of Fabry’s disease. Lancet Neurol.

[25] Feigin VL, Lawes CM, Bennett DA, Anderson CS (2003). Stroke epidemiology: a review of population-based studies of incidence, prevalence, and case-fatality in the late 20th century. Lancet Neurol.

[26] Politei J, Schenone AB, Burlina A, Blanco M, Lescano S, Szlago M, Cabrera G (2014). Vertebrobasilar dolichoectasia in Fabry disease: the earliest marker of neurovascular involvement?. J Inborn Errors Metab Screen.

[27] von Scheidt W, Eng CM, Fitzmaurice TF, Erdmann E, Hubner G, Olsen EG, Christomanou H, Kandolf R, Bishop DF, Desnick RJ (1991). An atypical variant of Fabry’s disease with manifestations confined to the myocardium. N Engl J Med.

[28] Takenaka T, Teraguchi H, Yoshida A, Taguchi S, Ninomiya K, Umekita Y, Yoshida H, Horinouchi M, Tabata K, Yonezawa S, Yoshimitsu M, Higuchi K, Nakao S, Anan R, Minagoe S, Tei C (2008). Terminal stage cardiac findings in patients with cardiac Fabry disease: an electrocardiographic, echocardiographic, and autopsy study. J Cardiol.

[29] Germain DP (2001). A new phenotype of Fabry disease with intermediate severity between the classical form and the cardiac variant. Contrib Nephrol.

[30] DeVeber GA, Schwarting GA, Kolodny EH, Kowall NW (1992). Fabry disease: immunocytochemical characterization of neuronal involvement. Ann Neurol.

[31] Bolsover FE, Murphy E, Cipolotti L, Werring DJ, Lachmann RH (2014). Cognitive dysfunction and depression in Fabry disease: a systematic review. J Inherit Metab Dis.

[32] Kolodny E, Fellgiebel A, Hilz MJ, Sims K, Caruso P, Phan TG, Politei J, Manara R, Burlina A (2015). Cerebrovascular involvement in Fabry disease: current status of knowledge. Stroke.

